# Gene expression regional differences in human subcutaneous adipose tissue

**DOI:** 10.1186/s12864-017-3564-2

**Published:** 2017-02-23

**Authors:** Angelina Passaro, Maria Agata Miselli, Juana Maria Sanz, Edoardo Dalla Nora, Mario Luca Morieri, Rossella Colonna, Rado Pišot, Giovanni Zuliani

**Affiliations:** 1 Azienda Ospedaliero Universitaria di Ferrara, Ferrara, Italy; 2 Department of Medical Sciences, Section of Internal Medicine and CardioRespiratory, University of Ferrara, Ferrara, Italy; 3 Science and Research Centre, University of Primorska, Koper, 6000 Slovenia

**Keywords:** Obesity, Microarray, Adipose tissue, SAT, VAT, Homeobox genes

## Abstract

**Background:**

Accumulation of visceral adipose tissue (VAT) is clearly associated with an increased risk of obesity-related diseases and all-cause mortality, whereas gluteal subcutaneous fat accumulation (g-SAT) is associated with a lower risk. The relative contribution, in term of cardiovascular risk, of abdominal subcutaneous adipose tissue (a-SAT) is still controversial with studies showing both a detrimental effect and a protective role.

Animal and in vitro studies demonstrated that adipocytes from visceral and subcutaneous depots have distinct morphological, metabolic and functional characteristics. These regional differences have a key role in the pathogenesis of obesity-related diseases. There is recent evidence that differentiation between upper-body and lower-body adipose tissues might be under control of site-specific sets of developmental genes, such as Homebox (HOX) genes, a group of related genes that control the body plan of an embryo along the anterior-posterior axis. However, the possible heterogeneity between different subcutaneous regions has not been extensively investigated.

Here we studied global mRNA expression in g-SAT and a-SAT with a microarray approach. RNA was isolated from g-SAT and a-SAT biopsy, from eight healthy subjects, and hybridized on RNA microarray chips in order to detect regional differences in gene expression.

**Results:**

A total of 131 genes are significantly and differently (>1.5 fold change, *p* < 0.05) expressed in a-SAT and g-SAT. Expression profiling reveals significant differences in expression of several HOX genes. Interestingly, two molecular signature of visceral adipocyte lineage, homebox genes HOXA5 and NR2F1, are up-regulated in a-SAT versus g-SAT by a 2.5 fold change.

**Conclusions:**

Our study shows that g-SAT and a-SAT have distinct expression profiles. The finding of a different expression of HOX genes, fundamental during the embryo development, suggests an early regional differentiation of subcutaneous adipose depots. Moreover, the higher expression of HOXA5 and NR2F1, two molecular signatures of visceral adipocytes, in a-SAT suggests that this subcutaneous adipose depot could be more similar to VAT than g-SAT.

Our data suggest that we should look at SAT as composed of distinct depots with possibly different impact in obesity associated metabolic complications.

**Electronic supplementary material:**

The online version of this article (doi:10.1186/s12864-017-3564-2) contains supplementary material, which is available to authorized users.

## Background

Obesity, defined as abnormal or excessive fat accumulation, increases risks for multiple metabolic diseases, such as type 2 diabetes (T2DM) and cardiovascular disease (CVD) [1].

Adipose tissue is a highly specialized loose connective tissue able to store large quantities of triacylglycerol (triglycerides) and fat-soluble substances. On a cytological level, adipose tissue is heterogeneous: the main parenchymal cells are the white adipocytes. White adipocytes are characterized by a unilocular lipid droplet occupying 95% of the cell volume, and a ‘squeezed’ nucleus. Despite adipocytes represent ~90% of the tissue volume, other cells type are present in adipose tissue such as preadipocytes, endothelial cells, pericytes, multipotent stem cells and immune system cells (macrophages, T-cells, neutrophils, lymphocytes).

It is generally regarded as a tissue without a specific anatomy. However, there are increasing data supporting the idea that adipose depots are organized to form a large organ with discrete anatomy, specific vascular and nerve supplies, complex cytology, and high physiological plasticity [2]. This organ is made up of several depots located in two main compartments of the body: subcutaneous adipose tissue (SAT) and visceral adipose tissues (VAT). SAT represents over 80% of total body fat and is most commonly distinguished in abdominal (a-SAT), gluteal-femoral depots (g-SAT). VAT, which is mostly associated with digestive organs, includes omental, mesenteric and epiploic adipose tissue depots. The adipose organ contributes to many fundamental biological functions: thermogenesis, lactation, immune responses and obviously energy balance and energy substrates partitioning.

More than 50 years ago J. Vague observed that not only fat mass but also adipose tissue distribution is clearly linked to CVD risk. Central obesity, characterized by an increase in VAT and abdominal SAT, confers increased risk of CVD, insulin resistance, T2DM and even all-cause mortality. On the other hand peripheral obesity, characherized by preferential accumulation of gluteo-femoral fat is associated with lower risk and may be protective [3–6].

At an anatomical level, different depots differ in cellular composition, microvasculature, innervation, metabolic characteristics, extracellular matrix composition [7]. Moreover different studies showed a different pattern of adipokines secretion and endocrine function between abdominal and lower-body adipose [8–14]. However, the relative contribution of abdominal subcutaneous adipose depot to increased cardiovascular is still controversial [15, 16].

The heterogeneity between different subcutaneous depots has also been investigated: abdominal subcutaneous adypocytes differ from femoral ones in term of proliferation [17], differentiation [18] and fatty acid release and accumulation capacity [19, 20]. Different adrenergic stimulation response [21, 22] and sexual hormones influence [19, 23] have also been reported, while data on possible differences in insulin effect among different subcutaneous depots are inconclusive [24].

Several studies suggest that different fat depots could arise from distinct precursors, derived from mesenchymal stem cells, with inherently different proliferative and adipogenic properties [25]. Gene expression profiling has identified different molecular signature of adipocyte lineage commitment. These genes, such as nuclear transcriptional receptor protein coding gene NR2F1 (Nuclear Receptor subfamily 2 group F member 1 or Coup-TF1), seem to play a major role in early cell-fate determination [26]. Similarly, another study underpinned that the profound functional differences between the upper-body and lower-body adipose tissues might be under control of site-specific sets of developmental genes, such as Homebox (HOX) genes, a group of related genes that control the body plan of an embryo along the anterior-posterior axis [27].

Here we studied global mRNA expression in gluteal and abdominal adipose tissues from eight healthy subjects, with a microarray approach, in order to detect regional differences in gene expression.

## Results

The anthropometric and metabolic characteristics of participants are shown in Table 1.

**Table 1:** Characteristics of all participants

Characteristic	Mean ± SD
Age (years)	60 ± 3
BMI (Kg/mq)	25.0 ± 3.0
FM (%)	21.4 ± 6.5
Total-C (mg/dl)	218.6 ± 24.2
Triglycerides (mg/dl)	147.9 ± 72
HDL-C (mg/dl)	43.7 ± 5.8
LDL-C (mg/dl)	145.3 ± 25.2

**Fig. 1:**
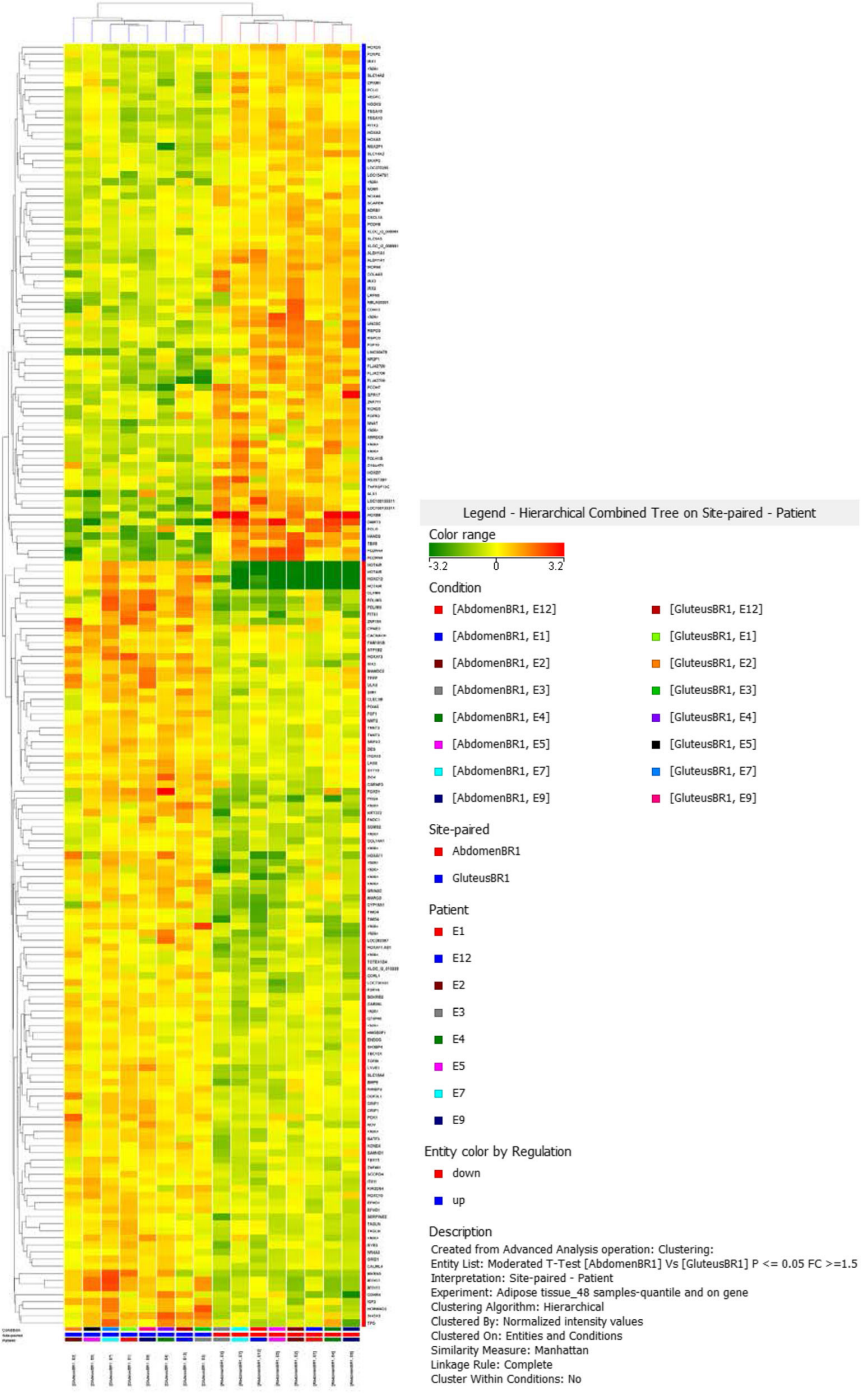
Gene clustering in abdominal and gluteal adipose tissue

In the microarray analysis, 42.405 probes were detected in abdominal and gluteal adipose tissues. Considering 1.5 fold change as lower limit, a total of 181 probes were differentially expressed between the abdominal and gluteal depot, corresponding for a total of 131 coding genes; 49 genes were up-regulated in abdominal versus gluteal adipose tissue (Table 2). Most of the expression differences were modest, >80% in 1.5–3 fold change range. Hierarchical clustering analysis of gene expression profiles in subcutaneous abdominal and gluteal adipose tissues of these 8 healthy subjects is represented in Fig. 1.

**Table 2:** Differential gene expression between abdominal and gluteal adipose tissue depots

Down-regulated in abdomen vs gluteus			Up-regulate in abdomen vs gluteus		
Gene	FC	Description	Gene	FC	Description
**HOXC12**	**45,90**	**Homo sapiens homeobox C12 (HOXC12), MRNA [NM_173860]**	DMRT3	13,75	Homo sapiens doublesex and mab-3 related transcription factor 3 (DMRT3), MRNA [NM_021240]
PDLIM3	5,02	Homo sapiens PDZ and LIM domain 3 (PDLIM3), transcript variant 1, MRNA [NM_014476]	**HOXB8**	**9,55**	**Homo sapiens homeobox B8 (HOXB8), MRNA [NM_024016]**
PDLIM3	4,50	Homo sapiens PDZ and LIM domain 3 (PDLIM3), transcript variant 1, MRNA [NM_014476]	HAND2	7,57	Homo sapiens heart and neural crest derivatives expressed 2 (HAND2), MRNA [NM_021973]
MYH11	3,89	Homo sapiens myosin, heavy chain 11, smooth muscle (MYH11), transcript variant SM2B, MRNA [NM_001040113]	PDZRN4	6,77	Homo sapiens PDZ domain containing ring finger 4 (PDZRN4), transcript variant 2, MRNA [NM_013377]
FOXD1	3,66	Homo sapiens forkhead box D1 (FOXD1), MRNA [NM_004472]	PDZRN4	6,02	Homo sapiens PDZ domain containing ring finger 4 (PDZRN4), transcript variant 1, MRNA [NM_001164595]
MKRN3	3,65	Homo sapiens makorin ring finger protein 3 (MKRN3), MRNA [NM_005664]	TBX5	5,55	Homo sapiens T-box 5 (TBX5), transcript variant 1, MRNA [NM_000192]
PRG4	3,63	Homo sapiens proteoglycan 4 (PRG4), transcript variant A, MRNA [NM_005807]	PCDH7	3,92	Homo sapiens protocadherin 7 (PCDH7), transcript variant a, MRNA [NM_002589]
**HOXA11**	**3,62**	**Homo sapiens homeobox A11 (HOXA11), MRNA [NM_005523]**	ALX1	3,77	Homo sapiens ALX homeobox 1 (ALX1), MRNA [NM_006982]
MYH11	3,62	Homo sapiens myosin, heavy chain 11, smooth muscle (MYH11), transcript variant SM1B, MRNA [NM_001040114]	PCLO	3,64	Homo sapiens piccolo (presynaptic cytomatrix protein) (PCLO), transcript variant 1, MRNA [NM_033026]
**HOXA13**	**3,46**	**Homo sapiens homeobox A13 (HOXA13), MRNA [NM_000522]**	ALDH1A1	3,08	Homo sapiens aldehyde dehydrogenase 1 family, member A1 (ALDH1A1), MRNA [NM_000689]
PITX1	3,44	Homo sapiens paired-like homeodomain 1 (PITX1), MRNA [NM_002653]	NNAT	3,03	Homo sapiens neuronatin (NNAT), transcript variant 1, MRNA [NM_005386]
OLFM4	3,07	Homo sapiens olfactomedin 4 (OLFM4), MRNA [NM_006418]	ALDH1A1	2,95	Homo sapiens aldehyde dehydrogenase 1 family, member A1 (ALDH1A1), MRNA [NM_000689]
SIX2	2,97	Homo sapiens SIX homeobox 2 (SIX2), MRNA [NM_016932]	FGF10	2,93	Homo sapiens fibroblast growth factor 10 (FGF10), MRNA [NM_004465]
ZNF154	2,91	Homo sapiens zinc finger protein 154 (ZNF154), MRNA [NM_001085384]	UNC5C	2,82	Homo sapiens unc-5 homolog C (C. elegans) (UNC5C), MRNA [NM_003728]
SHOX2	2,89	Homo sapiens short stature homeobox 2 (SHOX2), transcript variant 1, MRNA [NM_003030]	CDH12	2,82	Homo sapiens cadherin 12, type 2 (N-cadherin 2) (CDH12), MRNA [NM_004061]
KRT222	2,79	Homo sapiens keratin 222 (KRT222), MRNA [NM_152349]	RSPO3	2,70	Homo sapiens R-spondin 3 (RSPO3), MRNA [NM_032784]
CYP19A1	2,75	Homo sapiens cytochrome P450, family 19, subfamily A, polypeptide 1 (CYP19A1), transcript variant 2, MRNA [NM_031226]	**NR2F1**	**2,67**	**Homo sapiens nuclear receptor subfamily 2, group F, member 1 (NR2F1), MRNA [NM_005654]**
CDHR4	2,73	Homo sapiens cadherin-related family member 4 (CDHR4), MRNA [NM_001007540]	**HOXA5**	**2,56**	**Homo sapiens homeobox A5 (HOXA5), MRNA [NM_019102]**
ZIC4	2,69	Homo sapiens Zic family member 4 (ZIC4), transcript variant 3, MRNA [NM_032153]	IRX2	2,52	Homo sapiens iroquois homeobox 2 (IRX2), transcript variant 1, MRNA [NM_033267]
TPO	2,52	Homo sapiens thyroid peroxidase (TPO), transcript variant 5, MRNA [NM_175722]	COL4A5	2,45	Homo sapiens collagen, type IV, alpha 5 (COL4A5), transcript variant 2, MRNA [NM_033380]
HORMAD2	2,50	Homo sapiens HORMA domain containing 2 (HORMAD2), MRNA [NM_152510]	ZNF711	2,34	Homo sapiens zinc finger protein 711 (ZNF711), MRNA [NM_021998]
IGF2	2,44	Homo sapiens insulin-like growth factor 2 (somatomedin A) (IGF2), transcript variant 1, MRNA [NM_000612]	C19orf71	2,34	Homo sapiens chromosome 19 open reading frame 71 (C19orf71), MRNA [NM_001135580]
TIMD4	2,44	Homo sapiens T-cell immunoglobulin and mucin domain containing 4 (TIMD4), transcript variant 1, MRNA [NM_138379]	FOLH1B	2,33	Homo sapiens folate hydrolase 1B (FOLH1B), MRNA [NM_153696]
MARCO	2,36	Homo sapiens macrophage receptor with collagenous structure (MARCO), MRNA [NM_006770]	RSPO3	2,29	Homo sapiens R-spondin 3 (RSPO3), MRNA [NM_032784]
LYVE1	2,23	Homo sapiens lymphatic vessel endothelial hyaluronan receptor 1 (LYVE1), MRNA [NM_006691]	LRFN5	2,28	Homo sapiens leucine rich repeat and fibronectin type III domain containing 5 (LRFN5), MRNA [NM_152447]
GRIN2C	2,16	Homo sapiens glutamate receptor, ionotropic, N-methyl D-aspartate 2C (GRIN2C), MRNA [NM_000835]	TSGA10	2,24	Homo sapiens testis specific, 10 (TSGA10), transcript variant 1, MRNA [NM_025244]
SIM1	2,14	Homo sapiens single-minded homolog 1 (Drosophila) (SIM1), MRNA [NM_005068]	IRX2	2,22	Homo sapiens iroquois homeobox 2 (IRX2), transcript variant 1, MRNA [NM_033267]
NOV	2,07	Homo sapiens nephroblastoma overexpressed gene (NOV), MRNA [NM_002514]	FGFR2	2,20	Homo sapiens fibroblast growth factor receptor 2 (FGFR2), transcript variant 2, MRNA [NM_022970]
BMP5	2,07	Homo sapiens bone morphogenetic protein 5 (BMP5), MRNA [NM_021073]	SLC14A2	2,17	Homo sapiens solute carrier family 14 (urea transporter), member 2 (SLC14A2), transcript variant 1, MRNA [NM_007163]
CCRL1	2,06	Homo sapiens chemokine (C-C motif) receptor-like 1 (CCRL1), transcript variant 1, MRNA [NM_178445]	CPXM1	2,11	Homo sapiens carboxypeptidase X (M14 family), member 1 (CPXM1), transcript variant 1, MRNA [NM_019609]
MAMDC2	2,05	Homo sapiens MAM domain containing 2 (MAMDC2), MRNA [NM_153267]	PITX2	2,10	Homo sapiens paired-like homeodomain 2 (PITX2), transcript variant 2, MRNA [NM_153426]
ULK4	2,03	Homo sapiens unc-51-like kinase 4 (C. elegans) (ULK4), MRNA [NM_017886]	**HOXA3**	**2,10**	**Homo sapiens homeobox A3 (HOXA3), transcript variant 2, MRNA [NM_153631]**
CSRNP3	2,03	Homo sapiens cysteine-serine-rich nuclear protein 3 (CSRNP3), transcript variant 1, MRNA [NM_001172173]	PCLO	2,07	Homo sapiens piccolo (presynaptic cytomatrix protein) (PCLO), transcript variant 2, MRNA [NM_014510]
FNDC1	2,01	Homo sapiens fibronectin type III domain containing 1 (FNDC1), MRNA [NM_032532]	TSGA10	2,07	Homo sapiens testis specific, 10 (TSGA10), transcript variant 1, MRNA [NM_025244]
TIMD4	1,94	Homo sapiens T-cell immunoglobulin and mucin domain containing 4 (TIMD4), transcript variant 1, MRNA [NM_138379]	ARRDC5	2,07	Homo sapiens arrestin domain containing 5 (ARRDC5), MRNA [NM_001080523]
PCK1	1,94	Homo sapiens phosphoenolpyruvate carboxykinase 1 (soluble) (PCK1), MRNA [NM_002591]	TNFRSF13C	2,06	Homo sapiens tumor necrosis factor receptor superfamily, member 13C (TNFRSF13C), MRNA [NM_052945]
ODF3L1	1,93	Homo sapiens outer dense fiber of sperm tails 3-like 1 (ODF3L1), MRNA [NM_175881]	WDR66	2,03	Homo sapiens WD repeat domain 66 (WDR66), transcript variant 1, MRNA [NM_144668]
KIR2DS4	1,93	Homo sapiens killer cell immunoglobulin-like receptor, two domains, short cytoplasmic tail, 4 (KIR2DS4), MRNA [NM_012314]	**HOXB7**	**1,96**	**Homo sapiens homeobox B7 (HOXB7), MRNA [NM_004502]**
BATF3	1,91	Homo sapiens basic leucine zipper transcription factor, ATF-like 3 (BATF3), MRNA [NM_018664]	FOXP2	1,93	Homo sapiens forkhead box P2 (FOXP2), transcript variant 4, MRNA [NM_148900]
**HOXC10**	**1,88**	**Homo sapiens homeobox C10 (HOXC10), MRNA [NM_017409]**	SLC14A2	1,86	Homo sapiens solute carrier family 14 (urea transporter), member 2 (SLC14A2), transcript variant 1, MRNA [NM_007163]
CAB39L	1,87	Homo sapiens calcium binding protein 39-like (CAB39L), transcript variant 1, MRNA [NM_030925]	KCND3	1,83	Homo sapiens potassium voltage-gated channel, Shal-related subfamily, member 3 (KCND3), transcript variant 1, MRNA [NM_004980]
RYR3	1,86	Homo sapiens ryanodine receptor 3 (RYR3), transcript variant 1, MRNA [NM_001036]	IRX1	1,72	Homo sapiens iroquois homeobox 1 (IRX1), MRNA [NM_024337]
GRID1	1,85	Homo sapiens glutamate receptor, ionotropic, delta 1 (GRID1), MRNA [NM_017551]	SKAP2	1,71	Homo sapiens src kinase associated phosphoprotein 2 (SKAP2), MRNA [NM_003930]
SERPINE2	1,84	Homo sapiens serpin peptidase inhibitor, clade E (nexin, plasminogen activator inhibitor type 1), member 2 (SERPINE2), transcript variant 1, MRNA [NM_006216]	ADRB1	1,7	Homo sapiens adrenergic, beta-1-, receptor (ADRB1), MRNA [NM_000684]
EFHD1	1,83	Homo sapiens EF-hand domain family, member D1 (EFHD1), transcript variant 1, MRNA [NM_025202]	CXCL14	1,70	Homo sapiens chemokine (C-X-C motif) ligand 14 (CXCL14), MRNA [NM_004887]
TPPP	1,82	Homo sapiens tubulin polymerization promoting protein (TPPP), MRNA [NM_007030]	VEGFC	1,59	Homo sapiens vascular endothelial growth factor C (VEGFC), MRNA [NM_005429]
CACNA1H	1,79	Homo sapiens calcium channel, voltage-dependent, T type, alpha 1H subunit (CACNA1H), transcript variant 1, MRNA [NM_021098]	PCDH9	1,54	Homo sapiens protocadherin 9 (PCDH9), transcript variant 1, MRNA [NM_203487]
SRPX2	1,79	Homo sapiens sushi-repeat containing protein, X-linked 2 (SRPX2), MRNA [NM_014467]	SLC5A3	1,53	Homo sapiens solute carrier family 5 (sodium/myo-inositol cotransporter), member 3 (SLC5A3), MRNA [NM_006933]
TBX15	1,78	Homo sapiens T-box 15 (TBX15), MRNA [NM_152380]	HOOK2	1,52	Homo sapiens hook homolog 2 (Drosophila) (HOOK2), transcript variant 1, MRNA [NM_013312]
ZNF461	1,78	Homo sapiens zinc finger protein 461 (ZNF461), MRNA [NM_153257]			
EFHD1	1,78	Homo sapiens EF-hand domain family, member D1 (EFHD1), transcript variant 1, MRNA [NM_025202]			
COL14A1	1,77	Homo sapiens collagen, type XIV, alpha 1 (COL14A1), MRNA [NM_021110]			
SLC16A4	1,77	Homo sapiens solute carrier family 16, member 4 (monocarboxylic acid transporter 5) (SLC16A4), transcript variant 1, MRNA [NM_004696]			
KCNE4	1,76	Homo sapiens potassium voltage-gated channel, Isk-related family, member 4 (KCNE4), MRNA [NM_080671]			
TCTEX1D4	1,75	Homo sapiens Tctex1 domain containing 4 (TCTEX1D4), MRNA [NM_001013632]			
ITIH1	1,75	Homo sapiens inter-alpha-trypsin inhibitor heavy chain 1 (ITIH1), transcript variant 1, MRNA [NM_002215]			
CLEC3B	1,73	Homo sapiens C-type lectin domain family 3, member B (CLEC3B), MRNA [NM_003278]			
TNNT3	1,72	Homo sapiens troponin T type 3 (skeletal, fast) (TNNT3), transcript variant 3, MRNA [NM_001042780]			
CRIP1	1,72	Homo sapiens cysteine-rich protein 1 (intestinal) (CRIP1), MRNA [NM_001311]			
P2RY6	1,72	Homo sapiens pyrimidinergic receptor P2Y, G-protein coupled, 6 (P2RY6), transcript variant 2, MRNA [NM_176798]			
ATP1B2	1,71	Homo sapiens ATPase, Na+/K+ transporting, beta 2 polypeptide (ATP1B2), MRNA [NM_001678]			
TNNT3	1,7	Homo sapiens troponin T type 3 (skeletal, fast) (TNNT3), transcript variant 1, MRNA [NM_006757]			
TAGLN	1,69	Homo sapiens transgelin (TAGLN), transcript variant 1, MRNA [NM_001001522]			
TBC1D1	1,68	Homo sapiens TBC1 (tre-2/USP6, BUB2, cdc16) domain family, member 1 (TBC1D1), MRNA [NM_015173]			
BDKRB2	1,68	Homo sapiens bradykinin receptor B2 (BDKRB2), MRNA [NM_000623]			
LHX6	1,65	Homo sapiens LIM homeobox 6 (LHX6), transcript variant 1, MRNA [NM_014368]			
NR4A3	1,64	Homo sapiens nuclear receptor subfamily 4, group A, member 3 (NR4A3), transcript variant 3, MRNA [NM_173200]			
CRIP1	1,64	Homo sapiens cysteine-rich protein 1 (intestinal) (CRIP1), MRNA [NM_001311]			
TAGLN	1,64	Homo sapiens transgelin (TAGLN), transcript variant 1, MRNA [NM_001001522]			
SYT15	1,64	Homo sapiens synaptotagmin XV (SYT15), transcript variant b, MRNA [NM_181519]			
SAMHD1	1,63	Homo sapiens SAM domain and HD domain 1 (SAMHD1), MRNA [NM_015474]			
FAM181B	1,62	Homo sapiens family with sequence similarity 181, member B (FAM181B), MRNA [NM_175885]			
SGMS2	1,6	Homo sapiens sphingomyelin synthase 2 (SGMS2), transcript variant 1, MRNA [NM_152621]			
RIMBP3	1,6	Homo sapiens RIMS binding protein 3 (RIMBP3), MRNA [NM_015672]			
DES	1,57	Homo sapiens desmin (DES), MRNA [NM_001927]			
TGFBI	1,55	Homo sapiens transforming growth factor, beta-induced, 68 kDa (TGFBI), MRNA [NM_000358]			
SCCPDH	1,54	Homo sapiens saccharopine dehydrogenase (putative) (SCCPDH), MRNA [NM_016002]			
NMT2	1,54	Homo sapiens N-myristoyltransferase 2 (NMT2), MRNA [NM_004808]			
ITGA10	1,54	Homo sapiens integrin, alpha 10 (ITGA10), MRNA [NM_003637]			
PDIA5	1,54	Homo sapiens protein disulfide isomerase family A, member 5 (PDIA5), transcript variant 1, MRNA [NM_006810]			
CALML4	1,53	Homo sapiens calmodulin-like 4 (CALML4), transcript variant 1, MRNA [NM_033429]			
ENDOG	1,52	Homo sapiens endonuclease G (ENDOG), nuclear gene encoding mitochondrial protein, MRNA [NM_004435]			

Bold data was used to drive attention to the presence of many HOX genes, in our gene list. Underlined data was used to identify genes involved in type 2 diabtes pathogenesis

We identified several HOX genes expression differences between gluteal and abdominal depots (bold in Table 2). Mean fold change for HOX gene group was 8.88 (range from 1.88 to 45.90), compared to 2.84 mean fold change of entire group of differentially expressed genes. HOXA3, HOXA5, HOXB7, HOXB8, were up-regulated in the abdominal depot, while HOXA11, HOXA13, HOXC10, HOXC12 were up-regulated in gluteal adipose tissue, with HOXC12 expression being almost 50 times overexpressed in gluteal vs abdominal adipose tissue.

NR2F1 was up-regulated in abdominal vs gluteal adipose tissue (FC 2.67).

In silico analysis with bioinformatics database support (DAVID gene functional classification tool) showed that 18 out of 131 coding genes have been previously involved in type 2 diabetes pathogenesis (underlined in Table 2): TBX5, COL4A5, PITX2, FOXP2, SKAP2, ADRB1, VEGFC, PCDH9 were up-regulated in the abdominal depot, while CYP19A1, IGF2, PCK1, TNNT3, TBC1D1, BDKRB2, TAGLN, DES, TGFBI, NMT2 were up-regulated in gluteal adipose tissue.

## Discussion

Adipose tissue is considered as the largest endocrine organ in humans, and includes numerous discrete anatomical depots. There is evidence that different adipose tissue depots have different morphology, physiology and adipokine profiles. Although depot differences in adipocyte metabolism and endocrine function are clearly important in etiology of obesity related diseases, the relative contribution of VAT compared to abdominal subcutaneous is still controversial. Moreover, there is little evidence of in vivo or in vitro differences between different subcutaneous adipose depot.

The advent of new technology that allows the characterization of entire transcriptomes has permitted to look from another perspective to adipose tissue depots heterogeneity and led to the hope that the properties of adipose tissue and differences between adipose tissue depots might be revealed to help discover new therapeutic avenues.

Indeed, expression profiling has revealed significant differences in expression of hundreds of genes between different depots of adipose tissue in both rodents and humans [28–30], particularly developmental and patterning genes involved in cell differentiation, organogenesis, antero-posterior or dorso-ventral patterning. These findings not only have contributed to help explain the distinct impact of these depots on the development of metabolic complications but also have suggested possible differences in developmental origin of these fat cells [31].

Adipose tissue has a mesodermal origin. A layer of cells between the primitive endoderm and ectoderm migrates and spreads along the antero-posterior and dorso-ventral axes of the developing embryo generating the axial, intermediate, lateral plate, and paraxial mesoderm. Each of these embryologic tissues eventually gives rise to local adipose tissue. Vertebrate embryonic patterning and evolution of mesodermal tissues such as fat are controlled by several conserved developmental signaling systems. The mesenchimal stem cell gives origin to an early precursor, the adipoblast, which develops into committed preadipocytes that under appropriate stimuli differentiate into mature adipocytes of different types.

In more recent studies, the differences in gene expression pattern have been shown to persist even after in vitro differentiation of preadipocytes, suggesting that the differences are independent of extrinsic factors and that different adipocyte progenitors are programmed through epigenetic modulation during early development, participating in determining functional differences observed between different adipose tissue depots [27].

The most frequently observed differences in gene expression involve HOX genes, a subset of Homeobox genes. A Homeobox is a DNA sequence, around 180 base pairs long, found within genes that are involved in the regulation of patterns of anatomical development (morphogenesis) in animals, fungi and plants. These genes encode Homeodomain protein products that are transcription factors sharing a characteristic protein fold structure that binds DNA. Through the DNA-recognition properties of the Homeodomain, Homeoproteins are believed to regulate the expression of targeted genes and direct the formation of many body structures during early embryonic development. Many Homeodomain proteins induce cellular differentiation by initiating the cascades of coregulated genes required to produce individual tissues and organs. Thus, Homeobox genes are critical in the establishment of body axes during embryogenesis. The HOX genes in humans are organized in four chromosomal clusters: HOXA, HOXB, HOXC and HOXD.

In a 2006 study from Gesta et al., gene expression profiling has revealed that intraabdominal (visceral) adipocytes express higher levels of HOXA5, HOXA4, HOXC8 and NR2F1, whereas subcutaneous fat has higher levels of HOXA10, and HOXC9, and in most cases, these differences are observed in both rodents and humans. Similar differences in development gene expression are observed in preadipocytes isolated from different adipose depots of rodents. These differences in gene expression are large in magnitude (up to 1000-fold), appear to be intrinsic, and persist during in vitro culture and differentiation, indicating that they are cell autonomous and independent of the tissue microenvironment. In addition, the authors have shown that one of these developmental genes (HOXA5) exhibit changes in expression that closely correlate with the extent of obesity (BMI) and the pattern of fat distribution (WHR).

In recent study from Karastergiou et al. substantial differences in HOX genes expression have been found between two different subcutaneous adipose tissue depots, gluteal and abdominal. In our study, we confirm significant gene expression differences between abdominal and gluteal SAT, as well seen in the hierarchical clustering analysis. We underline differences in expression of developmental and patterning genes such as HOX genes (HOXA3, HOXA5, HOXB7, HOXB8, HOXA11, HOXA13, HOXC10, HOXC12), as previously observed.

We have also noticed as some genes, that in previous studies have been proposed as moleculare signatures of VAT, such as HOXA5 and NR2F1 appear to be up-regulated in abdominal compared to gluteal SAT (g-SAT), suggesting a similarity between VAT and abdominal SAT (a-SAT).

Furthermore, the finding that a consistent number of genes differentially expressed between gluteal and abdominal adipose depots have been previously correlated to pathogenesys of type 2 diabetes enforces our hypotesis that these depots may have a different impact in obesity associated metabolic complications.

## Limitations of the study

Some limitations of the study must be underlined.

First, this research was conducted on a small sample of single sex subjects: males with an average age of 60 years old. Therefore our findings apply only to men in this specific age group.

Secondly, we adopted >1.5 fold change as significant threshold of differences between the two groups, as previously seen in similar researches, however this increases the possibility of false positive.

## Conclusions

Our study demonstrates that subcutaneous gluteal and abdominal adipose tissue depots have distinct expression profiles. The finding of a different expression of HOX genes, fundamental during the embryo development, suggests an early regional differentiation of SAT, while the higher expression of HOXA5 and NR2F1, two molecular signatures of visceral adipocytes, in a-SAT suggests that this subcutaneous adipose depot could be more similar to VAT compared to g-SAT.

In conclusion we suggest that we should look at SAT as composed of distinct depots with possibly different impact in obesity associated metabolic complications.

## Methods

Subjects enrolled in the study were partecipants the project PANGeA (Physical Activity and Nutrition for Good quality Ageing). Eight healthy middle aged men [age 60 ± 3 years, (57–65)] underwent subcutaneous tissue biopsy from abdomen and gluteus subcutaneous adipose tissue. Adipose tissue samples were obtained from each participant from both abdomen and gluteus. Briefly, the subject was instructed to hold in tension the muscles, so that the muscle and the fat pad were clearly recognizable. A fold from the upper outer quadrant of the buttock and from the abdomen was held between two fingers of one hand; subsequently a needle (16–17 gauge), connected to a vacutainer system, was inserted with an angle of about 45° in the fat pad. After the insertion of the needle, the vacutainer tube was pressed forward to connect the vacuum with the needle. The needle was then carefully pushed back and forth 2–3 times within the fat pad to gather the adipose tissue biopsy. Subsequently the needle was immediately introduced in a sterile tube and frozen in liquid nitrogen. Adipose tissue was extracted from the needle with lysis solution (Purezool, Bio Rad, Milan, Italy) and then disrupted and homogenized using a tissue ruptor (Qiagen, Milan, Italy). RNA was isolated using Aurum Total RNA Mini kit (Bio Rad) and stored at -80 °C until use.

All participants were medically examined prior study inclusion with an interview, routine blood analysis, and fitness battery tests. Exclusion criteria were: smoking; regular alcohol consumption; acute or chronic skeletal, neuromuscular, respiratory, metabolic and cardiovascular disease conditions. Participants were informed of the purpose, procedures and potential risk of the study before signing the informed consent. Anthropometric data were presented by body mass index and fat mass that was measured using bio impedance with a tetra-polar impedance-meter (BIA101, Akern, Florence, Italy). Blood samples were collected after an overnight fasting. Adipose tissue samples were collected by biopsy from abdomen and gluteus and immediately frozen in liquid nitrogen. Total RNA was obtained from frozen tissue samples by using the a lysis solution (Purezool, Bio Rad, Milan Italy) and successively disrupted and homogenizated using a tissue ruptor. RNA was isolated (Aurum Total RNA Mini kit, Bio Rad) and stored at −80 °C until use. RNA labeling and hybridization on microRNA microarray chips was performed as previously described [32]. Microarray results were analysed using GeneSpring GX software 7.3 (Agilent Technologies). Data files were pre-processed using the GeneSpring plug-in for Agilent Feature Extraction software results. Data transformation was applied to set all the negative raw values at 5.0, followed by on-chip and on-gene median normalization. Filtering on gene expression was applied so that probes expressed (flagged as Present) in at least one sample were kept and probes that did not change across all samples, identified as having a normalized expression always between median ± 1.5, were removed. Then, samples were grouped in accordance to their status and compared. Differentially expressed genes were selected as having a 1.5-fold difference between their geometrical mean expression in the two adipose tissue and a statistically significant *p* value (<0.05) by ANOVA (analysis of variance), followed by application of the Benjamini and Hoechberg correction for false-positive reduction. Differentially expressed genes were employed for the cluster analysis of samples, using the standard correlation as a measure of similarity.

Finally, the list of genes differently expressed was analyzed with bioinformatics database support (DAVID gene functional classification tool) to find potential functional-related gene groups and gene-disease associations.

RNA quality was assessed by the use of Agilent 2100 Bioanalyzer (Agilent Technologies). Low quality RNAs (RNA integrity number below 7) were excluded from microarray analyses. Labeled cRNA was synthesized from 500 ng of total RNA using the Low RNA Input Linear Amplification Kit (Agilent Technologies) in the presence of cyanine 3-CTP (Perkin-Elmer Life Sciences, Boston, MA). Hybridizations was performed at 65 °C for 17 h in a rotating oven. Full dataset is available to download as Additional file 1.

## Additional file

Additional file 1: Microarray complete dataset (normalized). (XLSX 7797 kb)
